# Breast cancer prevention with liquiritigenin from licorice through the inhibition of aromatase and protein biosynthesis in high-risk women’s breast tissue

**DOI:** 10.1038/s41598-023-34762-z

**Published:** 2023-05-30

**Authors:** Atieh Hajirahimkhan, Caitlin Howell, Elizabeth T. Bartom, Huali Dong, Daniel D. Lantvit, Xiaoling Xuei, Shao-Nong Chen, Guido F. Pauli, Judy L. Bolton, Susan E. Clare, Seema A. Khan, Birgit M. Dietz

**Affiliations:** 1 Division of Breast Surgery, Department of Surgery, Robert H. Lurie Comprehensive Cancer Center, Feinberg School of Medicine, Northwestern University, 303 E. Superior, 4-220, Chicago, IL 60611 USA; 2 Department of Physiology and Biophysics, College of Medicine, University of Illinois Chicago, Chicago, IL USA; 3 Department of Biochemistry and Molecular Genetics, The Louis A. Simpson and Kimberly K. Querrey Biomedical Research Center, Feinberg School of Medicine, Northwestern University, Chicago, IL USA; 4 University of Illinois Cancer Center, College of Medicine, University of Illinois Chicago, Chicago, IL USA; 5 UIC Center for Botanical Dietary Supplements Research, Pharmacognosy Institute and Department of Pharmaceutical Sciences, College of Pharmacy, University of Illinois Chicago, Chicago, IL USA; 6 Department of Medical and Molecular Genetics, College of Medicine, Indiana University, Indianapolis, IN USA

**Keywords:** Cancer prevention, Natural products

## Abstract

Breast cancer risk continues to increase post menopause. Anti-estrogen therapies are available to prevent postmenopausal breast cancer in high-risk women. However, their adverse effects have reduced acceptability and overall success in cancer prevention. Natural products such as hops (*Humulus lupulus*) and three pharmacopeial licorice (*Glycyrrhiza*) species have demonstrated estrogenic and chemopreventive properties, but little is known regarding their effects on aromatase expression and activity as well as pro-proliferation pathways in human breast tissue. We show that *Gycyrrhiza inflata* (GI) has the highest aromatase inhibition potency among these plant extracts. Moreover, phytoestrogens such as liquiritigenin which is common in all licorice species have potent aromatase inhibitory activity, which is further supported by computational docking of their structures in the binding pocket of aromatase. In addition, GI extract and liquiritigenin suppress aromatase expression in the breast tissue of high-risk postmenopausal women. Although liquiritigenin has estrogenic effects in vitro, with preferential activity through estrogen receptor (ER)-β, it reduces estradiol-induced uterine growth in vivo. It downregulates RNA translation, protein biosynthesis, and metabolism in high-risk women’s breast tissue. Finally, it reduces the rate of MCF-7 cell proliferation, with repeated dosing. Collectively, these data suggest that liquiritigenin has breast cancer prevention potential for high-risk postmenopausal women.

## Introduction

**Figure 1:**
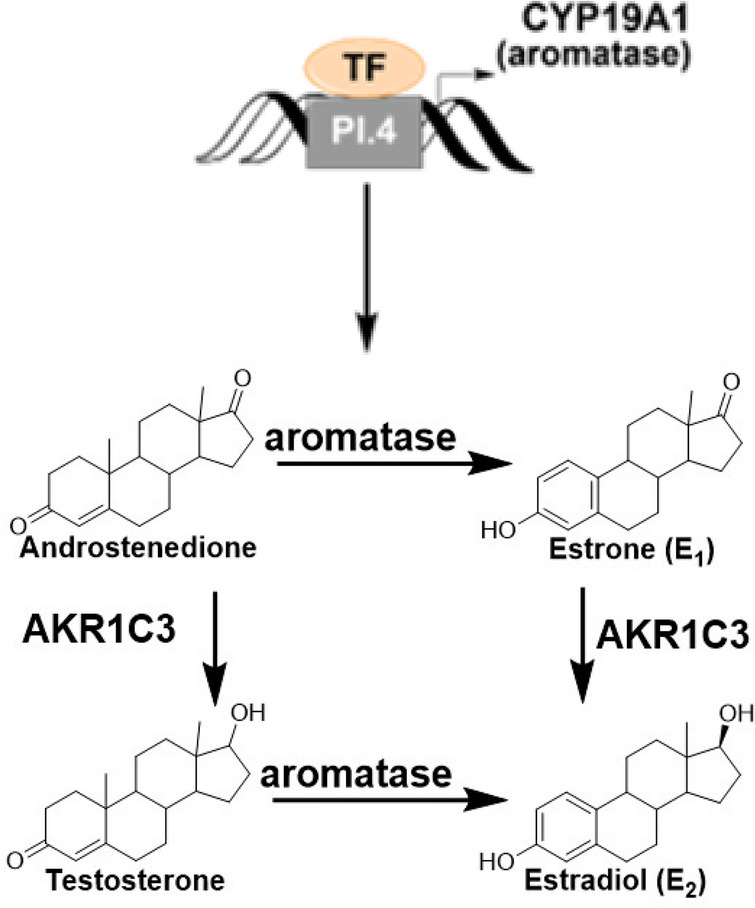
Estrogen biosynthesis. Aromatase is the rate limiting enzyme in estrogen biosynthesis. Suppression of its expression as well as inhibition of its activity decrease estrogen production, leading to reduced proliferation and tumor prevention, lowered hormone dependent proliferation, and reduced formation of genotoxic estrogen metabolites.

Breast cancer is the most common cancer and the second leading cause of cancer mortality in women, worldwide^1^. The majority of breast cancers are estrogen dependent or estrogen receptor positive (ER+)^2^, their risk increases with age, and they are the most common type in postmenopausal patients, despite the low level of circulating ovarian estrogens^2^. It is posited that estrogen biosynthesis in the adipose tissue, especially in the breast adipocytes plays an important role in sustaining exposure to estrogens and the development of ER + breast tumors after menopause^3^. Estrogen biosynthesis, whether in the ovaries or other organs/tissues, involves the conversion of the androgens, testosterone and androstenedione, to their respective estrogens, 17β-estradiol (E_2_) and estrone (E_1_), by the catalytic action of aromatase (CYP19A1), a member of the CYP450 enzymes family (Fig. 1). While additional steps catalyzed by enzymes such as aldoketoreductase 1C3 (AKR1C3), which converts E_1_ to E_2_, are important in estrogen biosynthesis, aromatase is the rate determining enzyme. Thus, its regulation and activity plays a key role in estrogen production and carcinogenesis^4^.

**Figure 2:**
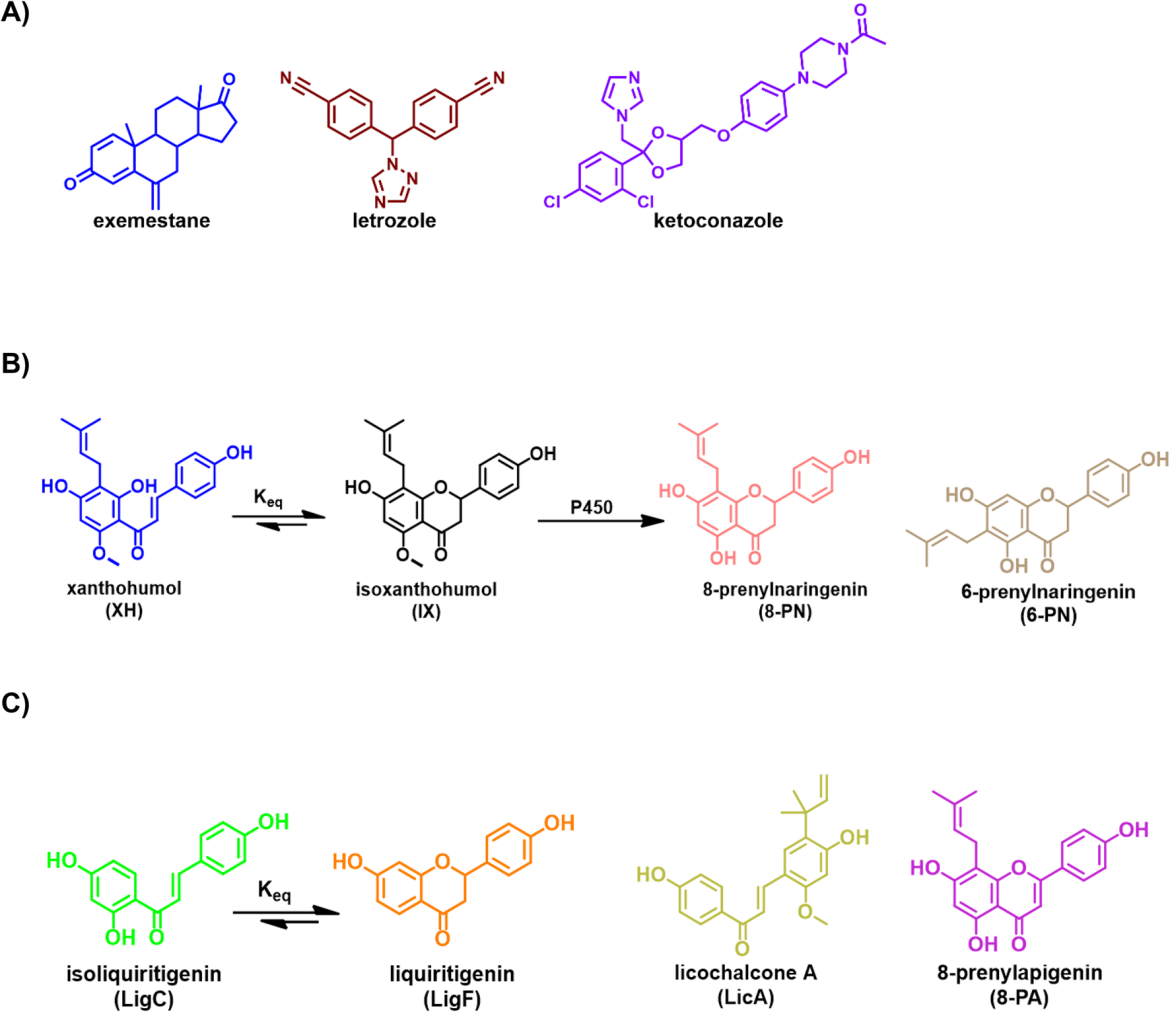
Chemical structures of (**A**) aromatase inhibitors, (**B**) hops bioactive compounds, and (**C**) licorice bioactive compounds.

Endocrine therapies such as aromatase inhibitors (AIs, Fig. 2A) are treatment options for postmenopausal women with ER + breast cancer and for premenopausal women with ER + breast cancers when combined with oophorectomy or ovarian suppression^5^. AIs such as letrozole (non-steroidal) and exemestane (steroidal) can effectively suppress estrogen levels in breast cancer patients and enhance survival^6^; they are also proven risk reducing agents in postmenopausal women at high risk for developing breast cancer^2^. While these drugs can reduce the risk of ER + breast cancer by 50–65%, they have had minimal impact on lowering the breast cancer incidence in the population at risk; mainly because few women who would potentially benefit from such interventions report taking them^2,5,7^. This is a result of limited acceptance and adherence due to side effects^8,9^ and to the reluctance of healthy individuals to accept drugs for prevention purpose^2,5,7^. Alternative interventions are needed with adequate efficacy yet lower toxicity that would lead to greater acceptability and better incorporation in women’s lives.

In search of such an option, we focused on botanicals such as hops (*Humulus lupulus*) and pharmacopeial licorice species (*Glycyrrhiza species)*, all of which are widely used by women for managing menopausal symptoms^10,11^. Being used as dietary supplements, these interventions are often part of daily intake and are used for extended periods of time^10,11^. Hops and licorice products have been reported to exhibit various effects which may protect against breast cancer occurrence. These include the suppression of estrogen genotoxic metabolism, enhancement of benign estrogen metabolism, induction of detoxification enzymes, prevention of DNA damage, as well as suppression of inflammation^10,12–26^. Together, the attributes of high acceptability by postmenopausal women, and the potential for creating a breast cancer preventive environment, point to a need for rigorous delineation of mechanisms that underlie the breast cancer prevention potential of these agents, for the development of more successful prevention strategies^10^.

Therefore, we report a systematic evaluation of the aromatase inhibitory effects of hops, and the three *Glycyrrhiza* species (*G. glabra*, GG; *G. uralensis*, GU; and *G. inflata*, GI) that are still used interchangeably as “licorice” botanical dietary supplements, and their major bioactive constituents. We also compared the binding of bioactive marker compounds of hops and licorice species to aromatase using computational approaches. We then studied changes in aromatase expression in postmenopausal women’s breast tissue microstructures exposed to these botanicals and their bioactive compounds. After observing improved results with the main phytoestrogens of hops (8-prenylnaringenin, 8-PN) (Fig. 2B) and licorice (liquiritigenin, LigF) (Fig. 2C), we assessed the uterine safety of these candidate agents. We knew from previous studies that 8-PN enhances uterine proliferation in rats, but the same data was not available for LigF. Therefore, we performed in vivo studies in rats; following the demonstration of a safe profile in the rats’ uterus, we conducted a total transcriptomic analysis in high-risk women’s breast tissue specimens exposed to LigF. To validate the transcriptomic results which suggested reduction in features of cell proliferation, such as protein synthesis, we performed live cell imaging in breast cancer cell lines exposed to LigF to monitor proliferation.

## Results

### Hops and licorice species as well as their major bioactive polyphenolic constituents inhibit aromatase

**Figure 3:**
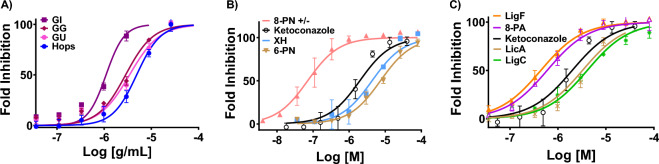
GI and the phytoestrogens from hops and licorice exhibit the most pronounced aromatase inhibition effect compared to the other tested agents. Aromatase supersomes were incubated with various concentrations of (**A**) GI, GG, GU, and hop extracts, (**B**) 8-PN, XH, 6-PN from hops, and (**C**) LigF, 8-PA, LicA, LigC from licorice for 40 min at 37 °C. Formation of a fluorescent metabolite was measured at Ex/Em of 409 nm/530 nm. Data represents mean ± SD of at least three independent measurements. Ketoconazole was used as the positive control.

Hops and the three licorice extracts (GG, GU, and GI) inhibited aromatase dose dependently (Figs. 3A, S1A). GI was the most potent extract with an IC_50_ value of 1 ± 0.14 µg/mL followed by GG (IC_50_ = 2.8 ± 0.12 µg/mL), GU (IC_50_ = 3.4 ± 0.12 µg/mL), and hops (IC_50_ = 4.9 ± 0.10 µg/mL) (Fig. 3A). Among the tested bioactive compounds from hops (Figs. 3B, S1B), 8-PN (Fig. 2B) was the most potent aromatase inhibitor with an IC_50_ value of 50 nM (Table 1), which was in the range of inhibitory potency of known aromatase inhibitors such as letrozole (IC_50_ ≈ 10–20 nM) ^27^. The rank order of IC_50_ values for the compounds from hops was 8-PN (50 nM) >  > XH (4.3 µM) > 6-PN (7.4 µM) (Table 1, Fig. 3B). Among the licorice compounds (Figs. 2C, 3C and S1C), LigF exhibited the highest inhibitory potency with an IC_50_ value of 430 nM, followed by 8-PA with an IC_50_ value of 590 nM. The rank order of IC_50_ values for the compounds from the three licorice species was LigF ≥ 8-PA > LicA (3.2 µM) ≥ LigC (4 µM) (Table 1). It should be noted that LicA is a marker compound specific for GI that has not been found in other species, whereas LigF is the common phytoestrogen in all the licorice species with total abundance of 4–12% w/w^28^, and 8-PA occurs in multiple *G.* species but is more concentrated in GI compared to other species^28–30^.

**Table 1:** Aromatase inhibition and estrogen receptor subtype (ERα, ERβ) binding affinities of compounds from hops and licorice species.

Compound	Aromatase inhibition (IC_50_, µM)	ERα binding (IC_50_, µM)	ERβ binding (IC_50_, µM)
ketoconazole	1.97 ± 0.1	N/A	N/A
xanthohumol (XH)	4.3 ± 0.6	N/A	N/A
8-prenylnaringenin (8-PN)	0.05 ± 0.004	0.060 ± 0.01^a^	0.16 ± 0.06^a^
6-prenylnaringenin (6-PN)	7.4 ± 0.3	N/A	N/A
isoliquiritigenin (LigC)	4.0 ± 0.3	16 ± 1^b^	7.8 ± 0.1^b^
liquiritigenin (LigF)	0.43 ± 0.02	> 200^b^	7.5 ± 0.5^b^
8-prenylapigenin (8-PA)	0.59 ± 0.08	0.1 ± 0.05^a^	0.03 ± 0.01^a^
licochalcone A (LicA)	3.2 ± 0.1	N/A	N/A

Data is presented as mean ± SD of at least three independent measurements.

^a^J Agric Food Chem. 2020, 68: 10651–10663.

^b^PLoS One, 2013, 8: e67947.

Overall, the most potent aromatase inhibitor compounds with inhibitory potencies in the nanomolar range were the phytoestrogens, 8-PN from hops followed by LigF, and 8-PA from licorice (Table 1). On the other hand, the non-estrogenic bioactive constituents such as XH and 6-PN from hops, as well as LicA and LigC from licorice did not exhibit aromatase inhibition effects at nanomolar concentrations (Table 1).

### Phytoestrogens from hops and licorice bind to aromatase similarly to letrozole

**Figure 4:**
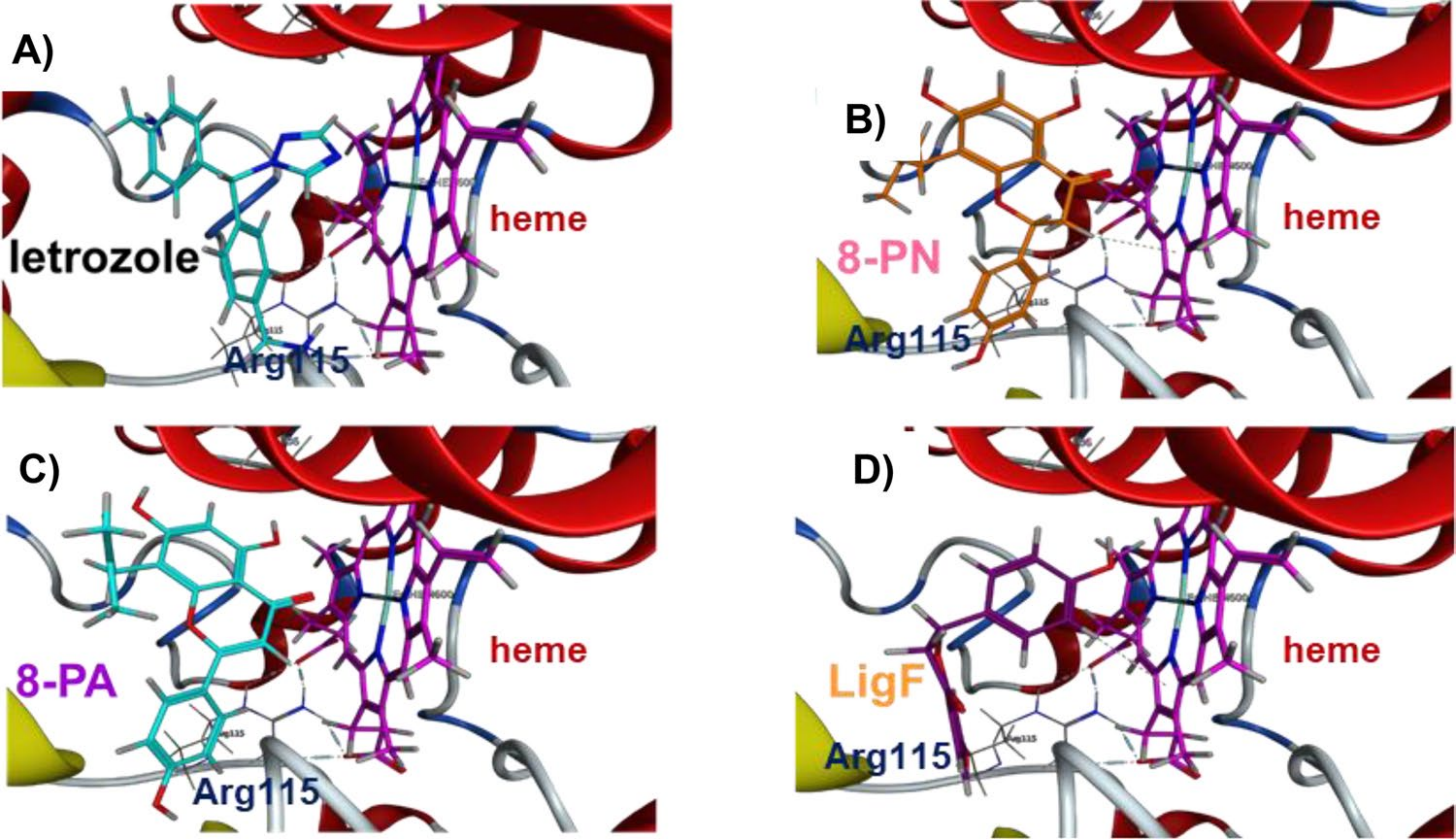
Phytoestrogens bind the active site of aromatase similar to letrozole, an established aromatase inhibitor. (**A**) letrozole (light blue), (**B**) 8-PN (orange), (**C**) 8-PA (light blue), and (**D**) LigF (dark purple) docked into aromatase (ribbon)-heme (fuchsia) complex in its most energetically favorable conformation.

The in vitro aromatase inhibition results (Figs. 3B, C, S1B, C) along with published data regarding the estrogen receptor binding of the compounds from hops and licorice^31,32^ (Table 1) suggested that the greater potency of phytoestrogens in aromatase inhibition might be due to a better binding to the aromatase binding pocket compared to the non-phytoestrogenic bioactive compounds. To further explore these effects, we performed in silico docking experiments using *a* model of aromatase and the chemical structures of the three phytoestrogens 8-PN, LigF, and 8-PA along with the non-estrogenic compounds XH, 6-PN, LicA, and LigC. We compared these to the binding of known aromatase inhibitors letrozole and exemestane (Fig. 2A). The phytoestrogens with high binding affinity bound in a similar location to the native ligand, androstenedione (A4) (Fig. S2A), as well as the potent aromatase inhibitors, letrozole (Figs. 4A, S2B), and exemestane (Fig. S2C), all of which interact with heme^33^. One of letrozole’s nitrile adjacent rings is located in a hydrophobic channel of the binding pocket (Fig. S2B). The hydrophobic prenyl group of the best binder, 8-PN, and the third best binder, 8-PA, extends in this same hydrophobic channel (Fig. 4B, C), contributing to their binding affinity. In its conformation with best binding affinity, 8-PA does not directly bind to heme in its most energetically favorable position (Fig. 4C). However, 8-PA does bind to heme in a less energetically favorable conformation (data not shown). If heme binding is important to aromatase inhibition, this less favorable conformation is a potential reason for the lower binding affinity. LigF in its best binding affinity directly binds to heme but the binding is not energetically as favorable as seen with 8-PN (Fig. 4D), but it is greater than that of 8-PA. LicA binds within the binding pocket but does not bind to heme in any of its top ten most energetically favorable poses (Fig. S3A). Although 6-PN did bind to the heme group (Fig. S3B), its prenyl group did not fall within the hydrophobic pocket (Fig. S4A). XH and LigC did not bind within the binding pocket (data not shown).

### Hops, licorice, and their bioactive polyphenols suppress aromatase expression in the breast tissue of high-risk postmenopausal women

For this experiment, we employed microstructures (Fig. S5) obtained from surgical high-risk breast tissue as described in our Materials and Methods section. These tissue preparations consist of various cell types of the breast such as adipocytes, glandular, myoepithelial, and endothelial cells. The composition of breast cell types is variable among different individuals, but the process of microstructure preparation enriches the mixture for epithelium. Given that microstructures have a larger starting tissue pieces that go through a more moderate and longer digestion, they tend to be more uniform compared to organoids. In addition, size exclusion filtering enhances their homogeneity. Overall, breast tissue microstructure cell composition consists of about 50% glandular cells, and 50% mixture of adipocytes, immune cells, and others^34,35^. Choosing suitable housekeeping genes and normalizing every donor’s experimental sample to her own control sample helps with reducing the variability for subsequent quantifications.

**Figure 5:**
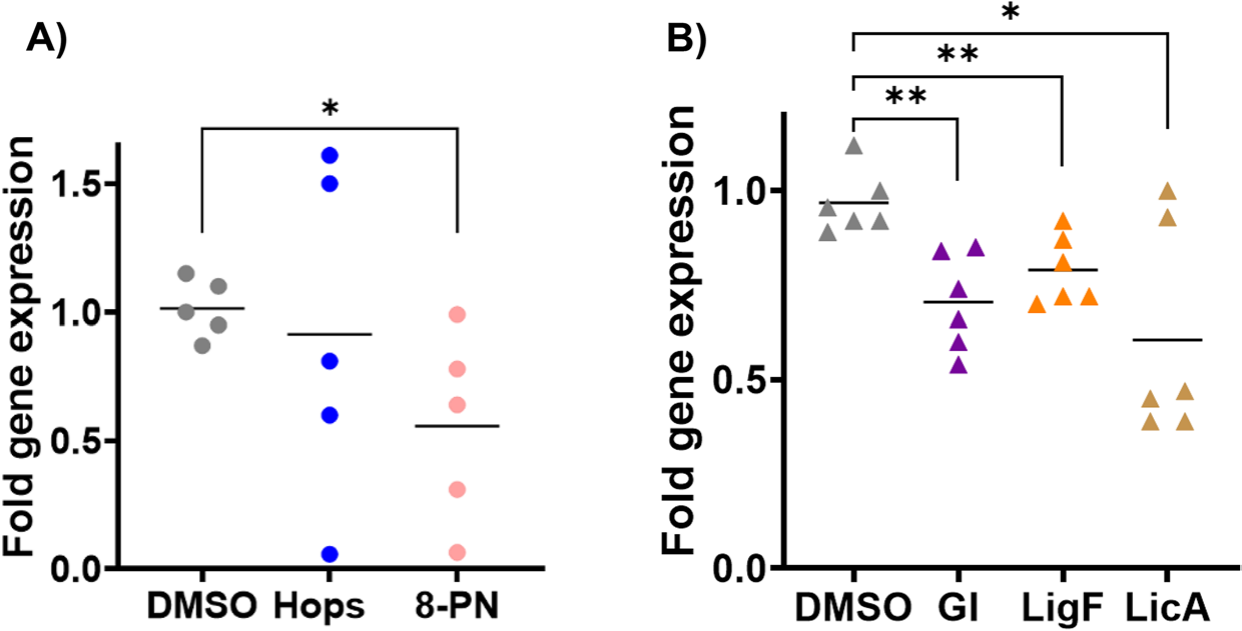
Hops and GI suppress the expression of CYP19A1 mRNA in high-risk breast tissue microstructures. Fresh surgically removed unaffected contralateral breast tissue from postmenopausal women undergoing double mastectomy due to unilateral breast cancer were processed to make microstructures using collagenase I, before culturing and treating them for 24 h with (**A**) hops (5 µg/mL), 8-PN (10 µM) vs. (**B**) GI (5 µg/mL), LigF (5 µM), LicA (5 µM). Subsequently, RNA extraction and qPCR of CYP19A1 was performed. The expression of CYP19A1 mRNA was calculated relative to the housekeeping gene RPLP0 and normalized to DMSO control. Data represents mean ± SD of three independent observations (*p* < 0.05).

In our work qPCR analysis of *CYP19A1* using the RNA extracted from breast microstructures of six high-risk postmenopausal women showed that the hops extract did not significantly change the base line expression levels of *CYP19A1* (Fig. 5A). However, the hop phytoestrogen, 8-PN, suppressed *CYP19A1* expression by a mean of 40% in 5 subject’s tissues (*p* < 0.025) when compared to the vehicle treated samples. In six subject’s tissues, GI extract suppressed baseline *CYP19A1* expression by nearly 30% (*p* < 0.025) (Fig. 5B). LigF, the key phytoestrogen from licorice, showed similar suppression levels (25%, *p* < 0.05), and LicA, a GI marker compound, suppressed the mean baseline *CYP19A1* expression by nearly 40% in the six subject’s tissues (*p* < 0.025).

### Liquiritigenin (LigF) the phytoestrogen from licorice with potent aromatase inhibition effect, suppresses E_2_-induced uterine growth

**Figure 6:**
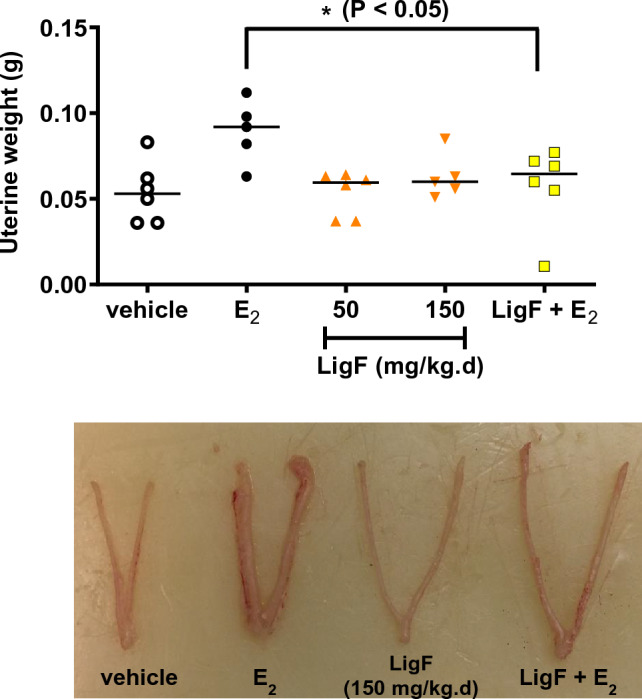
Liquiritigenin does not enhance uterine weight and suppresses E_2_-induced uterine growth. Female immature Sprague–Dawley rats (n = 6 per group) at 18 days of age were weaned and on day 19 of age received vehicle control, estradiol benzoate (1.6 µg/kg.day), LigF (50 mg/kg.day), LigF (150 mg/kg.day), and LigF (150 mg/kg.day) + estradiol benzoate (1.6 µg/kg.day) subcutaneously for 3 days. Animals were sacrificed on day 4 and their uterine weights were measured. The effects of LigF depicted in the accompanying picture of the animals’ uterine tissue are associated with LigF (150 mg/kg.day).

Based on the aromatase binding affinity and suppression of aromatase expression, and their potential clinical utility, it was reasonable to further evaluate LigF and 8-PN. However, 8-PN was already known to enhance uterine growth and deemed to be unsafe^31,36^. To determine if the estrogenic nature of LigF could pose a risk to estrogen responsive tissues such as uterus (which is the problem with 8-PN), we employed female immature Sprague–Dawley rats to assess estrogenic activity in vivo. Estradiol benzoate at 1.6 µg/kg.day significantly enhanced uterine weight (nearly doubled) compared to vehicle treated animals (Fig. 6). However, LigF at the physiologically relevant doses of 50 mg/kg.day and 150 mg/kg.day did not enhance uterine growth, significantly. Interestingly, in animals receiving estradiol benzoate, co-administration of LigF (150 mg/kg.day) suppressed E_2_-induced uterine growth, significantly. These data suggest that LigF might be a safe option to be further evaluated in human breast tissue.

### Liquiritigenin downregulates pathways associated with protein biosynthesis in high-risk postmenopausal breast tissues

**Figure 7:**
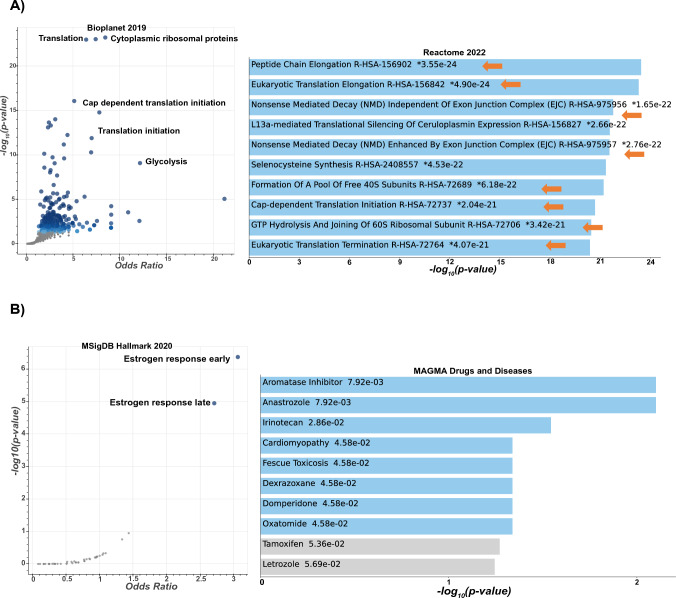
Transcriptomic analysis of high-risk breast tissue microstructures exposed to LigF suggests downregulation of protein synthesis. Breast tissue microstructures prepared from surgical contralateral unaffected breast of postmenopausal women with unilateral breast cancer were exposed to LigF (5 µM) for 24 h. RNA was extracted and total RNA sequencing was performed. Differential gene expression was evaluated using a patient-controlled approach. Significantly (adj *P* < 0.05) modulated genes were further analyzed by Enrichr to define modulated pathways and ontologies.

Utilizing microstructures obtained from six additional donors (different subjects from those used in qPCR experiments), RNA sequencing results showed that LigF (5 µM) significantly (adj *P* < 0.05) downregulated 2088 differentially expressed genes (Table S1). Gene Ontology and pathway analysis using several databases including (but not limited to) Bio Planet 2019 and Reactome 2022 (Fig. 7A) showed that these genes were mainly associated with ribosome assembly, regulation, and protein biosynthesis. Among these significantly downregulated genes are a large number of ribosomal protein genes (*RPLs, RPSs*)*,* eukaryotic translation initiation factors *EIF3s, EIF4s,* and eukaryotic translation elongation factors* (EEFs)*. Many of the known estrogen responsive genes including but not limited to *IL20, PDLIM3, LOXL2, and BARX2* were significantly downregulated (Table S1). LigF also significantly upregulated 1199 differentially expressed genes (adj *P* < 0.05) (Table S1), some of which, such as *TFF1, PgR, GREB1, EGR3, SUSD3,* are associated with estrogen responsive pathways as analyzed using Hallmark pathway analysis and shown in Fig. 7B. However, none of the top hit small molecules that shared similar transcriptomic data with LigF were among the estrogens or estrogenic compounds, although LigF had shown preferential ERβ activities in cell lines^32^. In these 6 women’s specimens *CYP19A1* expression was slightly (Log2FC: 0.6) enhanced; however, LigF shared significant (adj *P* < 0.05) transcriptomic similarities with aromatase inhibitors as represented by the analysis through MAGMA Drugs and Diseases database shown in Fig. 7B, which is consistent with our aromatase inhibition observations (Fig. 3C).

### Liquiritigenin retards cell proliferation of MCF-7 cells

**Figure 8:**
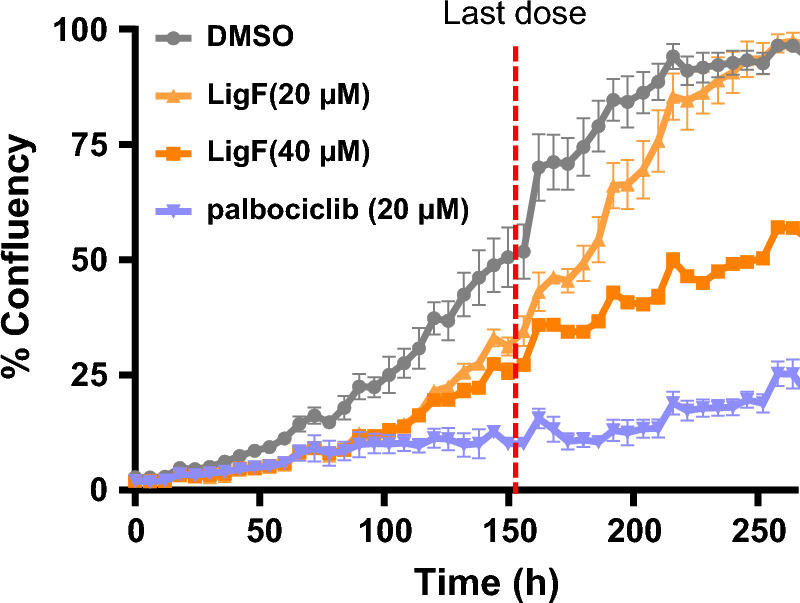
LigF retards breast cancer cell proliferation. MCF-7 cells were treated every 48 h with various concentrations of LigF for 6 days and observed for 11 days. Cell proliferation was monitored using live cell imaging by IncuCyte. Data represents the mean of at least two independent observations ± SEM.

Comparison of the gene expression signature between LigF-treated samples and the publicly available HMS LINCs KinomeScan suggested close similarities (adj *P* < 0.05) to the effects of seliciclib which is a broad cyclin dependent kinase (CDK) inhibitor. This observation suggested that LigF might have antiproliferative effects, in addition to its aromatase inhibition potential. We used a CDK 4/6 inhibitor, Palbociclib, as a positive control since this is in clinical use for treating patients with ER + breast cancer. LigF appears to be cytostatic; we did not detect cytotoxicity in the cells exposed to it. When a single dose of LigF was administered there was a clear reduction in cell proliferation for up to 48 h; however, if the dosing was not repeated the proliferation rate eventually caught up with the DMSO-treated cells. Therefore, we repeated the treatments every 48 h. We observed a profound suppression of proliferation at repeated doses of 20 µM and 40 µM and the effects continued to be significant until day 9 (Fig. 8). However, when treatment was halted after the third dose (six days, indicated with red dashed line, Fig. 8), cells in the LigF (20 µM) treatment group gradually caught up with the cell proliferation of DMSO-treated cells and reached full confluency on day 10. On the other hand, cells in the LigF (40 µM) treatment group maintained a significantly reduced proliferation rate until the end of the study (day 11), despite receiving the third/last dose of the treatment on day 6.

## Discussion

Breast cancer risk continues to rise after menopause despite the drastic decline in circulating ovarian estrogen^37^. Menopause is associated with increase in the adipose tissue fraction of the breast in both obese/overweight and lean women. Increased adiposity is known to lead to chronic low-grade inflammation, resulting in elevated local estrogen production in the breast stromal pre-adipocytes^3,38,39^. This E_1_/E_2_ enters the epithelial compartment in a paracrine manner, fueling the proliferation of epithelial cells and the genotoxic estrogen metabolism leading to carcinogenesis^40^. Compounds suppressing *CYP19A1* expression or directly inhibiting aromatase activity would therefore reduce local estrogen biosynthesis in the breast and serve to protect against breast cancer development.

Many postmenopausal women use botanicals such as hops and licorice species (GG, GI, GU), to relieve menopausal symptoms such as hot flashes^11^ and take these remedies for extended time periods as part of their daily routine. Research in this area has suggested that there may be a multitude of cancer prevention pathways modulated by these natural products making them suitable for further study. Aromatase inhibition is a known breast cancer preventive strategy for high-risk postmenopausal women; it has not been successful in prevention practice, however, due to the adverse side effects of currently available aromatase inhibitors. Establishing similar effects for natural products such as hops, licorice, and their bioactive compounds with substantially lower toxicity would lead to an alternative strategy with sufficient efficacy and potentially greater acceptability compared to the presently available interventions.

Our results suggest that hops, GG, GU, and GI contain aromatase inhibiting constituents and exhibit varying levels of inhibitory potential (Figs. 3A, S1). Of the extracts we tested, the highest inhibition potency was observed with GI (Figs. 3A, S1A) suggesting the presence of compound(s) with significant inhibitory potential in this extract. Data obtained with the pure compounds showed that the phytoestrogen LigF, which is abundant in all licorice species, and 8-PA, which is more abundant in GI, had aromatase inhibitory potencies at nanomolar concentrations (Figs. 3B, S1B, Table 1). LigC and the GI specific bioactive, LicA, exhibited only moderate inhibitory effects. Therefore, the observed aromatase inhibitory potency of GI likely relates to the presence of these constituents, in decreasing order: LigF, 8-PA, LigC, and LicA, (in addition to effects of other unknown compounds in the extract).

Compared to GI, hops exhibited a moderate aromatase inhibition (Figs. 3A, S1A), despite its potent phytoestrogen, 8-PN, exhibiting the highest aromatase inhibition effect compared to all the tested compounds (Figs. 3C, S1C). However, 8-PN has relatively low abundance in hops extract (0.33%), whereas its precursor XH, is a moderate aromatase inhibitor yet much more abundant constituent (32% of the extract). Conversion of XH to 8-PN does not happen under the conditions of in vitro aromatase inhibition assays. However, when the extract is ingested in vivo, 8-PN will form via IX as intermediate which is in equilibrium with XH (Fig. 2B), through the metabolic activities of cytochrome P450’s and gut microbiota, leading to potentially more pronounced aromatase inhibitory effects with hops extract in vivo. However, this needs to be explored in the future.

Binding of these phytoestrogens to the aromatase binding pocket was compared to known representatives of two classes of aromatase inhibitors (Fig. 2A), letrozole (non-steroidal) and exemestane (steroidal). These were evaluated computationally, using in silico models of aromatase (Fig. 5)^28,32,41^. These calculations suggest that the potent aromatase inhibitors, 8-PN from hops, and LigF and 8-PA from licorice, bind in the aromatase binding pocket similarly to the known inhibitors, letrozole and exemestane.

While both exemestane and letrozole block the binding of androstenedione via their binding to residues within aromatase’s binding pocket, exemestane irreversibly binds, while letrozole reversibly coordinates with heme iron and residues^33^. The compounds with high binding affinity bind in a similar location to letrozole and exemestane (Fig. 5). In their most energetically favorable conformation, 8-PN and LigF bind to the heme complex (Fig. 5). Past studies found that removal or blocking of the C4 carbonyl present in these compounds greatly reduced binding affinity, further suggesting the importance of heme interaction^42–44^.

Although letrozole’s heme interaction is known, the conformation of the rest of the molecule is less well-established. In this study, in its most favorable position, one of letrozole’s nitrile adjacent rings is located in a hydrophobic channel of the binding pocket (Fig. S2B) as documented in literature^45^. The hydrophobic prenyl group of the best binder, 8-PN, and the third best binder, 8-PA, extends in the area of this same hydrophobic channel (Figs. 5, S4). Likewise, past aromatase inhibition studies have found C8 prenylation of naringenin (8-PN) decreased the IC_50_ more than tenfold, while C6 prenylation led to poor aromatase binding affinity^12,46^. When combined with the results of the current study, these findings suggest that 8-PN and 8-PA may bind well due to their prenyl group falling within the hydrophobic channel when heme-binding occurs.

The compounds with microM IC_50_ values, XH, 6-PN, LigC, and LicA, neither bound within the binding pocket, nor interacted directly with heme, or had groups in energetically unfavorable positions. XH (data not shown) did not fall fully within the binding pocket when bound in its most energetically favorable position. Although falling within the binding pocket, LicA (Fig. S3A) and LigC (data not shown) did not directly bind to the heme group, whereas 6-PN did. However, the hydrophobic prenyl group of 6-PN was located in an unfavorable position within a hydrophilic region (Fig. S4A). While these computational results should be confirmed in a future structure–activity relationship study, they suggest that the phytoestrogens 8-PN from hops, and LigF and 8-PA from licorice species have the potential to act as natural aromatase inhibitors.

In addition to the direct inhibition of the enzyme activity, limiting estrogen production in the postmenopausal breast could also be achieved by suppressing the expression of the aromatase gene^3,47^, *CYP19A1*, which will in turn result in lower active enzyme levels and limited E_1_/E_2_ production. We studied how microstructures (S5) obtained from high-risk postmenopausal women’s breast tissue respond to these natural products. Microstructures are very similar to organoids but their size is slightly bigger due to their different method of generation to enhance uniformity and reproducibility. They contain a variety of breast cell types including pre-adipocyte fibroblasts capable of generating estrogens; they maintain the protein expression patterns, and the architectural features of the original tissue, and therefore better resemble physiological conditions^34^. These tissue preparations as well as organoids have been very useful in studying microenvironmental and stromal-epithelial interactions, although their uniformity, reproducibility, complexity, and structural accuracy need to be further improved^48^. We observed that the baseline levels of *CYP19A1* in vehicle-treated microstructures were high, and that the exposure to hops, 8-PN, GI, LigF, and LicA for 24 h did not affect the morphology of the microstructures in culture. However, 8-PN from hops, GI extract, LigF, and LicA decreased the expression of *CYP19A1* significantly (Fig. 5). These findings suggested the hypothesis that these natural products might have effects on regulating aromatase in cancer-free breast tissue and could be relevant to cancer prevention.

Although 8-PN from hops and LigF from licorice displayed aromatase inhibitory effects in our experiments, they are also well-known phytoestrogens. We therefore asked if their estrogenic nature could pose a risk for estrogen responsive tissues such as uterus. 8-PN is the most potent phytoestrogen known to date and has been frequently reported to have uterotrophic effects in animal models^31,36^. We, therefore, evaluated LigF for its uterine proliferation effects in immature female rats which is an established short-term model for assessing estrogenic activity in vivo^49^. The lack of uterine growth effects in LigF treated animals and the suppression of E_2_-induced uterine growth (Fig. 6) could be associated with several underlying mechanisms: (1) we have previously shown that LigF is a partial agonist of ER^32^, and therefore, can behave as an antiestrogen in the presence of E_2_, thus reducing proliferation, (2) LigF is suggested to have selective ERβ agonist activity, which can suppress ERα-dependent proliferation^32,50^, (3) LigF may modulate other pathways/targets leading to limiting proliferation. We concluded that LigF is a better candidate for further evaluation than 8-PN, and therefore, we assayed its effect on gene expression in additional women’s breast tissue. Our transcriptomic studies on additional microstructures exposed to LigF revealed that the modulation of estrogen responsive genes was not similar to estrogens or known estrogenic compounds and the transcriptomic signature of LigF in the breast was close to that of the aromatase inhibitors and broad CDK inhibitors (Fig. 7B). Moreover, pathways associated with translation, ribosome regulation, and metabolism were profoundly downregulated (Fig. 7A). These observations may explain the antiproliferative effects that we observed in MCF-7 cells when they were repeatedly dosed with various concentrations of LigF (Fig. 8). Interestingly, when the cells no longer received LigF (20 µM), they gained back their normal proliferation, an effect less evident with LigF (40 µM). This might suggest that repeated dosing of higher concentrations of LigF would better limit proliferation, but it needs to be assessed in the future in in vivo studies.

Overall, our data suggest that widely used women’s health botanicals such as hops, GG, GU, GI (Fig. 3A), and their phytoestrogens 8-PN, LigF, and 8-PA (Figs. 2, 3B, C) have aromatase inhibition effects, while 8-PN, GI, LigF, and LicA also suppress aromatase expression (Fig. 5) in breast microstructures from high-risk postmenopausal women. LigF appears to be the most promising candidate among the tested agents, since it inhibits aromatase, does not promote uterine growth, limits the protein translation machinery, resembles aromatase and CDK inhibitor signatures in ex vivo experiments using human breast samples, and reduces proliferative activity in breast cancer cell lines. Additional studies using high-risk breast tissue and in vivo evaluations are under way to extend and elaborate on these observations.

## Conclusions

**Figure 9:**
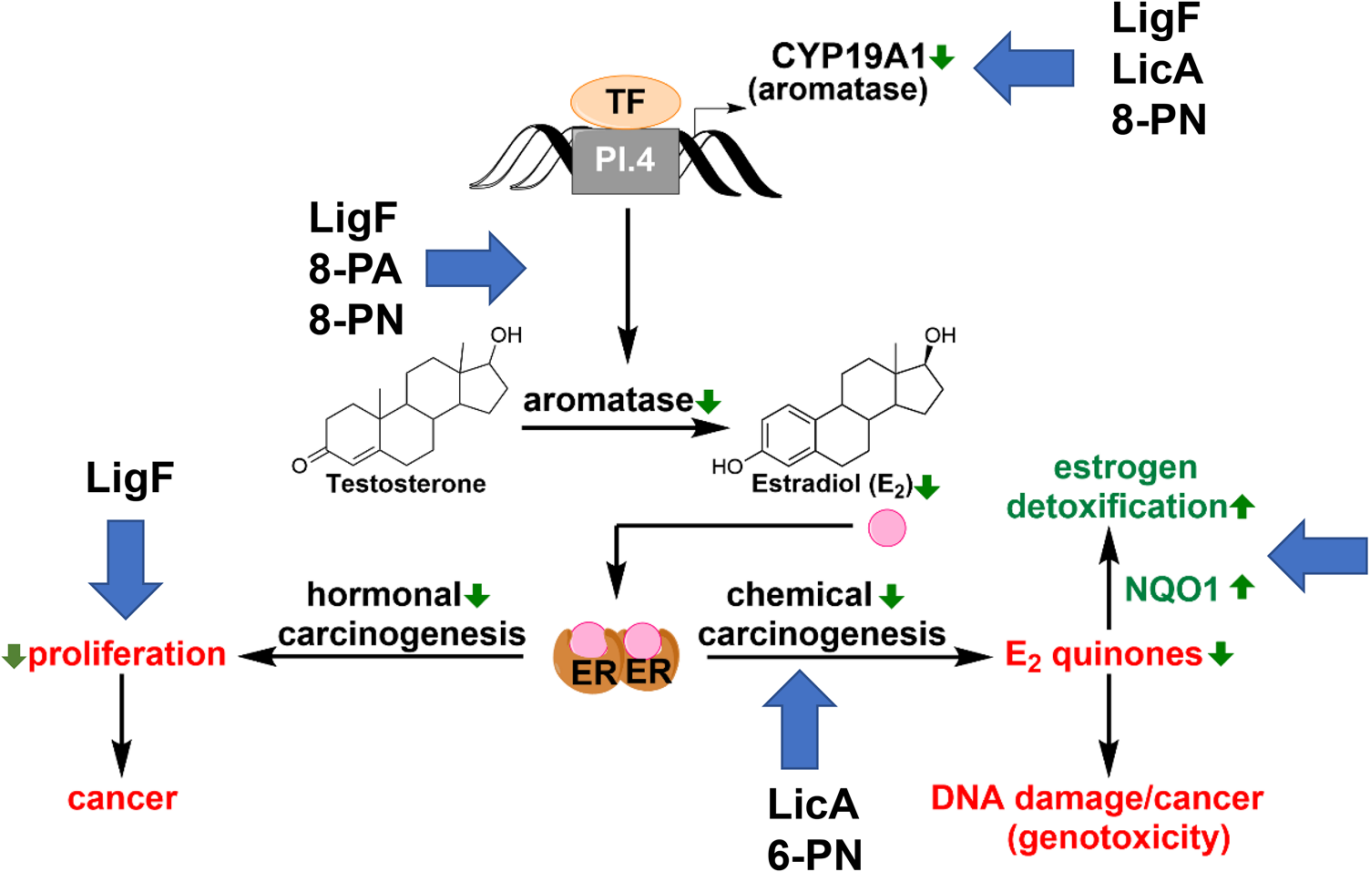
Summary of the effects of bioactive compounds from hops and licorice species on the pathways associated with estrogen carcinogenesis. Blue arrows show the points where the designated bioactive compounds exert an effect. Green arrows demonstrate the direction of the change in the designated biological activity as a function of the designated bioactive compounds.

We have evaluated popular natural products from licorice and hops for their aromatase inhibition potential and have further evaluated the most promising candidate, LigF, derived from licorice. We have shown that LigF has a multitude of breast cancer preventive potential (Fig. 9), resembling aromatase and CDK inhibitor drugs in women’s breast tissue with no uterotrophic effects in animal models. Our results in addition to the previous findings about these natural products (Fig. 9) also provide the impetus for botanical standardization efforts aimed at enriching GI extracts with LigF for cancer prevention purposes. Such interventions are highly likely to be acceptable to women, thus overcoming a major barrier to impactful and successful breast cancer prevention interventions^2^.

## Material and methods

### Chemicals and materials

All chemicals and reagents were purchased from Sigma-Aldrich (St. Louis, MO), unless otherwise indicated. MammoCult media kit, heparin, and hydrocortisone were purchased from Stem Cell Technologies (Vancouver, BC. Canada). F12-K nutrient mix (Kaighn’s) medium was acquired from Gibco (Dublin, Ireland). Fetal bovine serum (FBS) was purchased from Atlanta Biologicals (Norcross, GA). Collagenase I was purchased from Sigma-Aldrich (St. Louis, MO). Isoliquiritigenin (LigC) and liquiritigenin (LigF) were acquired from ChromaDex (Irvine, CA). 8-prenylapigenin (8-PA) was purchased from Ryan Scientific Inc. (Mount Pleasant, SC). Licochalcone A (LicA), 8-prenylnaringenin (8-PN), and 6-prenylnaringenin (6-PN) were acquired from Sigma-Aldrich (St. Louis, MO) and their purity assessed independently. Xanthohumol (XH) was isolated from hops (*Humulus lupulus*) as described previously^51^. CYP19/MFC high throughput screening kit was purchased from Corning (Corning, NY). Direct-Zol RNA prep kit was acquired from Zymoresearch (Irvine, CA). TRIzol was obtained from Invitrogen (Waltham, MA). PCR reagents, primers, and master mix were purchased from Integrated DNA Technologies (Coralville, IA).

### Preparation and characterization of plant extracts

Plant materials were sourced in accordance with good botanical research practices. The ethanol extract of botanically authenticated strobili of *Humulus lupulus* was dispersed on diatomaceous earth and extracted with CO_2_ yielding spent hops. The spent hop extract was obtained from Hopsteiner (Mainburg, Germany, and New York, NY) and standardized to the prenylated polyphenol marker compounds 6-PN, 8-PN, IX, and XH as previously described^41,52^. Briefly, standardization involved characterization by LC-UV, LC–MS/MS, and quantitative ^1^H NMR (qHNMR). The same extract has been used in a Phase I clinical trial in postmenopausal women^53^. The concentrations of the four marker compounds in this extract were 1.2% 6-PN, 0.33% 8-PN, 0.99% IX, and 32% XH. The extracts of the three different licorice species (*Glycyrrhiza glabra* L., *G. uralensis* Fisch. ex DC., and *G. inflata* Batalin, Fabaceae) were the chemically characterized methanol extracts of the respective dried and DNA-identified licorice root powders, as described previously^14,29,32,54^. The purity of the investigated pure compounds was determined by quantitative 1D ^1^H NMR using the 100% method^55^ and yielded the following purity percentages (in % w/w): LicA 96.1% (ratio trans/cis = 93/7), LigF 96.6%, LigC 98.6%, 8-PN 95.9%, 6-PN 98.5%, 8-PA 98.8%, and XH 96.5%.

### Aromatase inhibition assay

To evaluate the aromatase inhibitory potential of hops, licorice species, and their bioactive compounds, the Corning® Supersomes™ P450 Inhibition Kit CYP19/MFC high throughput inhibition assay was used. The protocol of the kit, which is based on the effects of the tested agents on the conversion of the fluorescent substrate, 7-methoxy-4-trifluoromethyl coumarin (MFC) to 7-hydroxy-4-trifluoromethyl coumarin (HFC) by aromatase, was followed. Briefly, in a 96 well plate, NADPH cofactor mix containing cofactors, G6PDH, control protein, and water were incubated with various concentrations of the test agents and ketoconazole (positive control) in 37 °C for 10 min. Then the enzyme/substrate mixture containing the aromatase enzyme and MFC were added to the pre-incubated mixture and were incubated at 37 °C for 30 min before stopping the enzymatic reaction with the stop solution. The fluorescent signal was evaluated at the Excitation/Emission of 409 nm/530 nm and was corrected for the potential innate fluorescence of the tested extracts and compounds.

### In silico docking analysis

In silico docking analysis was performed to investigate the interactions of the isoflavones with the aromatase-heme complex. The binding site of heme-bound human placental aromatase in complex with androstenedione was obtained from the Protein Data Bank (PDB ID: 3EQM) and uploaded to Molecular Operating Environment (MOE; Chemical Computing Group, version 2016.0208). All water molecules and androstenedione were removed, and the MOE QuickPrep function optimized the aromatase-heme complex with standard settings, which included structural error correction, partial charges calculation, hydrogen addition (Protonate3D), and a minimization of pocket and ligand residues within 8 Å of the ligand. The resulting complex was selected as receptor for all docking studies. Compounds were docked with triangle matcher placement with London dG scoring and induced fit refinement with GBVI/WSA dG scoring. The images are shown with the docked compound in its best-fit position with pi bonds shown as dashed lines. Molecular surface showing hydrophobicity and lipophilicity of the binding site was generated by MOE (supplementary data). 2D ligand interaction diagrams were generated by MOE ligand interaction function (Supplementary Data).

### Human breast microstructure preparation and treatment

To prepare breast microstructures the protocol of Brisken’s group was used with some minor modifications^34,35^. The unaffected contralateral breast tissues of postmenopausal women who underwent bilateral mastectomy due to incident unilateral breast cancer were obtained while fresh and diced to approximately 5 mm pieces before digesting at 37 °C with 2% collagenase I in F12-K nutrient mix (Kaighn’s) medium, overnight. After the completion of digestion, the tissue was centrifuged at 250×*g* for 5 min and the supernatant was discarded. The pellet was washed with phosphate buffered saline and was mixed and cultured in MammoCult media supplemented with 0.2% heparin and 0.5% hydrocortisone. After 24 h incubation at 37 °C the treatments were mixed with fresh MammoCult media and added to the microstructures.

### Ethics declarations

Institutional Review Board (IRB) approval (IRB STU00202331) was obtained from Northwestern University prior to obtaining informed consents from patients and collecting samples. All experiments were conducted in accordance with the approved protocol and guidelines.

### RNA preparation and qPCR

After 24 h incubation with the treatments, the microstructure pellet of the first 6 donors’ samples were prepared separately (not pooled) and washed with HBSS. Trizol was added to each pellet and the protocol of Direct-Zol RNA prep kit was used to extract RNA. The protocol included a step of DNAse I treatment for removing DNA contamination of the RNA preparations. RNA was eluted in nuclease free water and the concentration was measured using Nano Drop 2000 spectrophotometry (Thermo Fisher). The Integrated DNA Technologies (IDT) protocol, pre-made aromatase (*CYP19A1*) gene primer, and the pre-made housekeeping *RPLP0* gene primer was used to perform qPCR using QuantStudio 12 K Flex Real Time PCR system.

### In vivo uterotrophic studies

We used the OECD guidelines and animal model for testing uterotrophic or estrogenic activity of compounds^49^. Female immature Sprague–Dawley rats were received at 11 days of age from Harlan (Indianapolis, IN) and while the moms were on phytoestrogen free diet, pups stayed with moms until day 18 when they were whined and were randomized into groups of 6: (i) control diet plus vehicle control (s.c. corn oil); (ii) control diet plus 17β-estradiol benzoate (1.6 µg/kg BW per day, s.c.); (iii) control diet plus LigF (50 mg/kg BW per day, s.c.); (iv) control diet plus LigF (150 mg/kg BW per day, s.c.); (v) control diet plus simultaneous administration of LigF (150 mg/kg BW per day, s.c.) and 17β-estradiol benzoate (1.6 µg/kg BW per day, s.c.). The animals were treated for 3 days every 24 h. Animal weight and food intake were evaluated daily. Animals were sacrificed by CO_2_ asphyxiation. Uterus was collected, trimmed of fat and connective tissue, and weighted.

### Ethics declarations

The animal study was approved by the Institutional Animal Care and Use Committee (IACUC) at the University of Illinois Chicago (protocol number 08-101). All the experiments were conducted in accordance with the approved protocol and guidelines. The authors complied with the ARRIVE guidelines.

#### RNA sequencing

Microstructures obtained from high-risk surgical breast tissues (explained above) of 6 additional subjects (different from the first 6 donors used in qPCR experiments), were exposed to LigF (5 µM) for 24 h. Individual RNA samples (not pooled) were prepared, and quality assessment was performed using Agilent Bioanalyzer and Qubit. Libraries were made using Roche KAPA Biosystems protocol (KAPA RNA Hyper Prep Kit Technical Data Sheet, KR1352-v4.17, Roche Corporate) and the quality was evaluated. Total RNA sequencing was performed with Illumina NovaSeq 6000 v1.5, 2 × 100 b, pair end with 40 million reading depth, followed by quality assessment, alignment, and data analysis to define differential gene expression, as described previously^56–58^. Significantly (adj *P* < 0.05) modulated genes were further analyzed with Enrichr to define highly modulated pathways.

### Cell proliferation

MCF-7 cells were seeded at density of 2.5 × 10^3^ cells/well in RPMI media supplemented with 10% FBS. Cells were placed in IncuCyte for live cell imaging every 6 h, and were monitored. When the cells reached 30% confluency the first doses of DMSO, various concentrations of LigF, or various concentrations of Palbociclib (positive control) were administered. Treatments were repeated every 48 h until day 6 and cells were monitored until day 12. Data was analyzed using Zoom software to quantify the images and were plotted as mean ± SD of at least two independent measurements.

#### Statistical analysis

Data were analyzed using GraphPad Prism 9 and were expressed as the means ± SD. For qPCR data the analysis was followed by unpaired t-test to express the difference between the two groups. A *p*-value of less than 0.025 was considered statistically significant. For the Animal study the differences between the means were evaluated using One way ANOVA followed by Tukey’s multiple comparison with a p-value significance of less than 0.05.

## Supplementary Information

Supplementary Information 1.

Supplementary Information 2.

## Data Availability

The transcriptomic datasets generated during and/or analyzed during the current study are available through the NCBI GEO repository and the accession number is GSE223524. The rest of the results are included in this published article (and its Supplementary information files).
