# Supplementary material for: Breast cancer prevention with liquiritigenin from licorice through the inhibition of aromatase and protein biosynthesis in high-risk women’s breast tissue
Source: Sci Rep. 2023 May 30;13:8734. doi: 10.1038/s41598-023-34762-z (PMC10229614; doi:10.1038/s41598-023-34762-z)
Supplement: Supplementary file 1 — Supplementary Information 1. [file 41598_2023_34762_MOESM1_ESM.docx]

**Supporting Information**

**Breast cancer prevention with liquiritigenin from licorice through the inhibition of aromatase and protein biosynthesis in high-risk women’s breast tissue.**

Atieh Hajirahimkhan^1*^, Caitlin Howell^2^, Elizabeth T. Bartom^3^, Huali Dong^4^, Daniel D. Lantvit^5^, Xiaoling Xuei^6^, Shao-Nong Chen^5^, Guido F. Pauli^5^, Judy L. Bolton^5,ϯ^, Susan E. Clare^1,^, Seema A. Khan^1^, Birgit M. Dietz^5^.

^1^ Division of Breast Surgery, Robert H. Lurie Comprehensive Cancer Center, Feinberg School of Medicine, Northwestern University, Chicago, IL.

^2^ Department of Physiology and Biophysics, College of Medicine, University of Illinois at Chicago, Chicago, IL.

^3^ Department of Biochemistry and Molecular Genetics, The Louis A. Simpson and Kimberly K. Querrey Biomedical Research Center, Feinberg School of Medicine, Northwestern University, Chicago, IL.

^4^ University of Illinois Cancer Center, College of Medicine, University of Illinois at Chicago, Chicago, IL.

^5^ UIC Center for Botanical Dietary Supplements Research, Pharmacognosy Institute and Department of Pharmaceutical Sciences, College of Pharmacy, University of Illinois at Chicago, Chicago, IL.

^6^ Department of Medical and Molecular Genetics, College of Medicine, Indiana University, Indianapolis, IN.

* Corresponding author

Ϯ In memory of Dr. Judy L. Bolton.

**Correspondence:** Dr. Atieh Hajirahimkhan, Ph.D., American Cancer Society postdoctoral fellow, Robert. H. Lurie Comprehensive Cancer Center, Department of Surgery, Feinberg School of Medicine, Northwestern University, 303 E. Superior, 4-220, Chicago, IL. 60611. Email: [atieh.hajirahimkhan@northwestern.edu](mailto:atieh.hajirahimkhan@northwestern.edu), Phone: 312-503-2109

**Conflicts of Interest and Source of Funding:** AH received an American Cancer Society postdoctoral fellowship 131667-PF-18-049-01-NEC, GFP, SNC, JLB, BMD received a grant P50 AT000155 by NCCIH and ODS, GFP received a grant U41 AT008706 (CENAPT), S. A. K. received Bramsen-Hamill Foundation funds. For the remaining authors none were declared.

**A)**

**B)**

**C)**

**Figure S1:** Relative activity of aromatase as a function of incubation with different concentrations of A) hops, and licorice extracts, B) hops bioactive compound, C) licorice bioactive compounds.


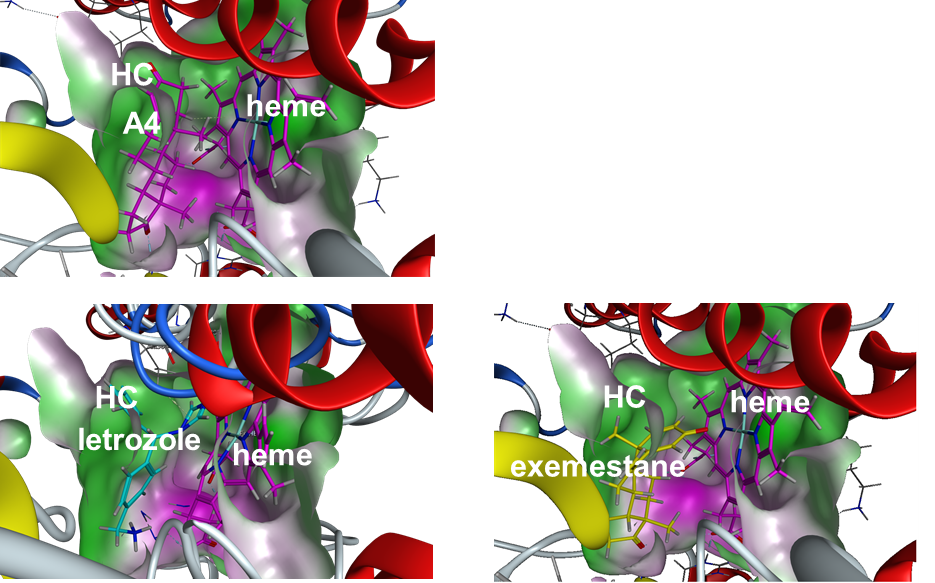


**C)**

**B)**

**A)**

**Figure S2:** A) Androstendione (A4), B) letrozole, and C) exemestane in the binding pocket of aromatase with heme present. Green surfaces represent hydrophobic regions; purple surfaces represent hydrophilic regions of the binding pocket. HC is the hydrophobic channel as discussed in the main text.

**A)**

*
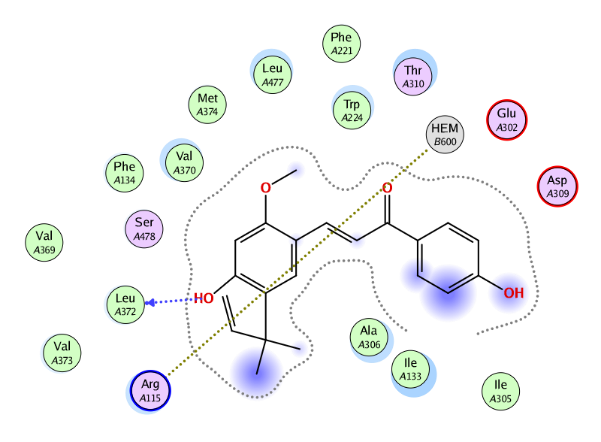
*

***
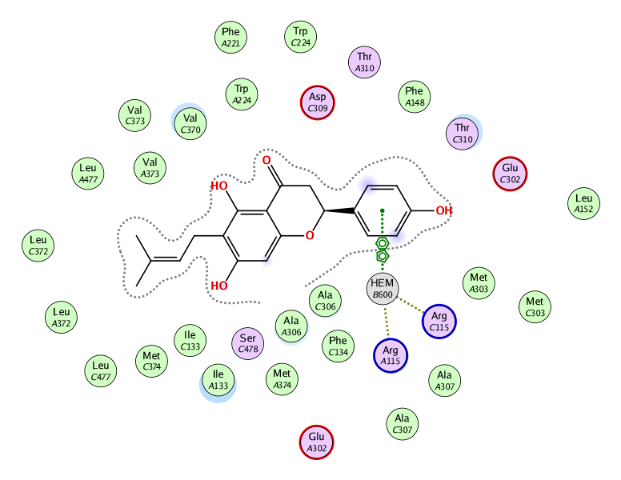
*B)**

**Figure S3:** Whereas LicA does not directly bind to heme (HEM) in the binding site of aromatase, 6-PN does: A) 2D interaction diagram of the most energetically favorable conformation of LicA; B) 2D overlay interaction diagram of top two most energetically favorable binding conformations of 6-PN bound to aromatase (3EQM).


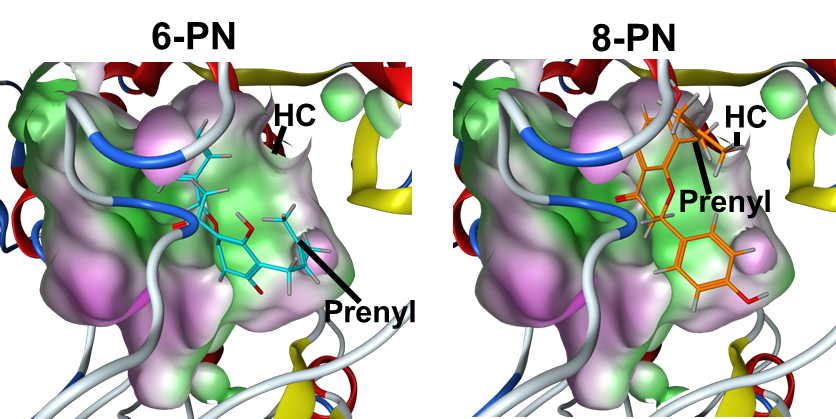


**B)**

**A)**

**Figure S4:** While the prenyl group of 6-PN does not fall within the hydrophobic channel (HC) of the aromatase protein, the same moiety of 8-PN does: A) 6-PN (light blue) vs. B) 8-PN (orange) docked into aromatase (ribbon)-heme (removed from picture) complex in its most energetically favorable conformation with hydrophobicity surface. Green surfaces are the hydrophobic region of binding pocket; purple surfaces are the hydrophilic regions.


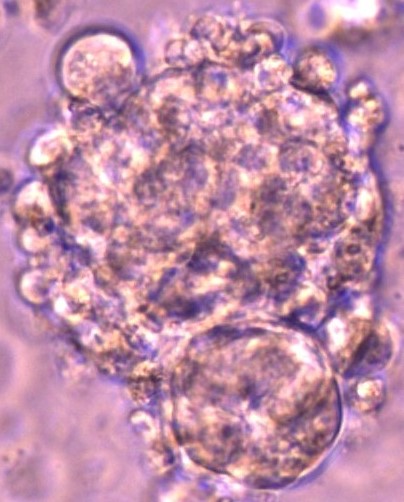


**Figure S5.** The 40X image of a microstructure prepared from the tumor free but at high-risk surgical breast tissue of a human subject. Breast microstructures are scaffold free tissue preparations obtained through slow and moderate digestion of fresh surgical breast tissue. They consist of various cell types present in the breast tissue such as epithelial, endothelial, immune cells, and adipocytes. They maintain the architectural features and the protein expression patterns of the original tissue.
